# From innovation to implementation: a global scoping review of artificial intelligence applications, governance, and challenges in humanitarian contexts

**DOI:** 10.7189/jogh.16.04359

**Published:** 2026-10-19

**Authors:** Mahima Jose, Fouzia Mueen, Junaid Qadir, Alaa Abd-alrazaq, Kwalombota Kwalombota, Diana Maddah

**Affiliations:** 1 Department of Public Health, College of Health Sciences, QU Health, Qatar University, Doha, Qatar; 2 College of Engineering, Qatar University, Doha, Qatar; 3 AI Centre for Precision Health, Weill Cornell Medicine-Qatar, Doha, Qatar; 4 United Kingdom Foreign, Commonwealth and Development Office, London, United Kingdom

**Keywords:** artificial intelligence, humanitarian setting, emergency management cycle, MENA region, governance, implementation

## Abstract

**Background:**

Artificial intelligence (AI) is increasingly being explored as a tool for supporting humanitarian action through hazards and crisis prediction, real time monitoring of evolving crisis conditions and humanitarian needs, resource allocation, and decision-making. However, evidence on how AI is implemented, evaluated, and governed across humanitarian contexts remains fragmented. In this scoping review, we mapped the global evidence on the application of AI in humanitarian settings, including its distribution across the emergency management cycle, deployment maturity, and evaluation. We also identified any governance considerations, as well as enabling factors for and challenges to its implementation, with particular attention to the Middle East and North Africa (MENA) region, Gulf Cooperation Council countries (GCC), and Qatar.

**Methods:**

The methodology of this scoping review followed the framework set by Arksey and O’Malley, which comprises the formulation of the research question, selection of records, data charting, and synthesis of findings. We searched six databases (ProQuest, IEEE Xplore, ScienceDirect, Embase, Web of Science, and Scopus), queried Google Scholar, and ran targeted grey literature searches through the webpages of major humanitarian organisations for records reporting on the application, implementation, or governance of AI in humanitarian contexts. We descriptively and narratively synthesised the extracted data.

**Results:**

We included 57 scholarly publications and 17 grey literature reports. Application of AI was most frequently reported in sudden-onset natural disasters, particularly floods and earthquakes, and was commonly used to support disaster monitoring, crisis informatics, search-and-rescue, logistics optimisation, hazard prediction, and geospatial analysis. Deep learning and machine learning were the most common model families. Across the emergency management cycle, most applications of AI focused on preparedness and response, while recovery was rarely addressed. Geographically, most evidence originated from Asia, while research from the MENA and GCC regions remained limited. Most reported systems remained prototypes or retrospectively validated models, with relatively few seeing operational deployment or field implementation. Governance issues were discussed more extensively in grey literature than in scholarly publications. Community participation, equity considerations, and formal governance frameworks were inconsistently reported.

**Conclusions:**

AI is increasingly being applied in humanitarian contexts, but it is heterogeneously distributed across regions, humanitarian contexts, and emergency management. Greater emphasis is needed on real-world deployment, implementation-focused evaluation, participatory approaches, and governance frameworks that support responsible, equitable, and context-sensitive AI adoption.

The combined impacts of climate change, armed conflicts, forced human migration, and public health emergencies have led to a rise in humanitarian crises, which now occur more frequently and with greater intensity and complexity. These events disrupt crucial infrastructure and essential services while destroying livelihoods, creating urgent needs that require global collaboration for resolution [1,2]. Here, we use the terms ‘humanitarian aid’, ‘humanitarian context’, and ‘humanitarian setting’ to describe responses to both natural hazards (such as floods and earthquakes) and human-induced crises (including conflict and displacement) occurring across the emergency management cycle (EMC) of mitigation, preparedness, response, and recovery [3].

Humanitarian aid began as short-term responses to humanitarian crises; however, many crises have now evolved into protracted humanitarian contexts that require sustained operational partnerships and long-term mission planning [4]. These changing dynamics create a need for more advanced analytical tools that can handle large-scale datasets and enable timely decision-making by humanitarian organisations.

Here, we define artificial intelligence (AI) as a broad set of computational approaches that support learning, prediction, classification, pattern recognition, automated analysis, and decision support in humanitarian contexts [5–7]. This includes machine learning (ML), deep learning (DL), natural language processing (NLP), computer vision, predictive analytics, geospatial AI, optimisation-based systems, and AI-enabled digital platforms used for tasks such as hazard prediction, damage assessment, displacement monitoring, resource allocation, crisis informatics, and humanitarian coordination [5–7]. Application of AI are increasingly being used for hazard prediction, damage assessment, displacement monitoring, resource allocation, and humanitarian coordination [5–7]. However, the extent to which these applications are operationally deployed, validated, and governed in humanitarian settings remains insufficiently documented.

Evidence from scholarly publications and organisational reports has not been consistently synthesised: while the former often emphasise algorithm development, predictive modelling, and technical performance, the latter more frequently describe practical tools, operational experience, governance concerns, and field implementation [6,8–13]. This has resulted in a fragmented evidence base in which technical, operational, ethical, legal, and governance-related dimensions of humanitarian AI are often examined separately. Previous reviews have addressed AI applications, technical capabilities, or ethical challenges in disaster and humanitarian settings, but have less consistently integrated evidence across the full EMC, deployment maturity, evaluation and validation stages, governance considerations, participation, equity-related themes, and implementation barriers [6,7,10–13]. To address these gaps, we designed a scoping review that would map the global evidence on AI applications in humanitarian contexts, while examining evidence from the Middle East and North Africa (MENA) region, Gulf Cooperation Council (GCC) countries, and Qatar as a regional sub-focus within the broader global literature. The MENA region, and especially GCC countries, provides an important regional lens because it faces overlapping humanitarian challenges, including conflict, displacement, climate-related hazards, and public health emergencies, while also pursuing rapid digital transformation and AI-related policy development [14,15]. The aforementioned GCC countries, especially Qatar, also play an important role in humanitarian funding, international assistance, and technology investment, creating a relevant context for examining how AI is being studied and applied in humanitarian settings [16]. Within the broader global scope of this review, we focused from the outset on the MENA region, particularly GCC countries and Qatar, as a regional area of interest, given its relevance to our objectives.

For this scoping review, we set three research questions that dealt with the operational application of AI, distribution across the EMC, AI model families, deployment maturity, evaluation and validation, governance considerations, enabling factors, and implementation barriers:

1. Which applications of AI have been reported on in humanitarian contexts globally, and specifically in the MENA region, GCC countries, and Qatar?
2. How are these applications distributed by humanitarian contexts, operational use cases, AI model families, emergency management phases, deployment maturity, and its evaluation ?
3. What governance considerations, enabling factors, implementation barriers, participation, and equity-related issues does the included evidence report on?

## METHODS

We designed this scoping review according to the methodological framework of Arksey and O’Malley [17] and report its findings per the PRISMA-ScR guidelines [18].

### Search Strategy

We developed our search strategy using the population – concept – context framework (Table S1 in the **Online Supplementary Document**), where we utilised both controlled vocabulary and free-text terms to find information about AI (*e.g.* ‘machine learning’, ‘deep learning’, ‘natural language processing’, ‘predictive analytics’) and humanitarian setting (*e.g.* ‘disaster management’, ‘humanitarian assistance’, ‘emergency management’). We examined AI applications in the global context, while the MENA- and GCC-related terms acted as sensitivity identifiers, rather than geographic filters. We included records from every geographic region if it fulfilled our inclusion criteria.

We queried ProQuest, IEEE Xplore, ScienceDirect, Embase, Web of Science, and Scopus, with the final searches through all six databases completed on 24 January 2026. Moreover, we conducted a supplementary Google Scholar search to identify potentially relevant records that may not have been captured through the six databases. The search used combinations of the main search concepts and terms derived from the primary database search strategy, including terms related to AI and humanitarian settings. We screened the records appearing on the first ten pages of Google Scholar results for eligibility. In terms of grey literature, we searched the databases and websites of key organisations: ReliefWeb, the World Bank, United Nations International Children’s Emergency Fund (UNICEF), International Federation of Red Cross and Red Crescent Societies (IFRC), OCHA, Humanitarian Data Exchange, the United Nations High Commissioner for Refugees (UNHCR), and the World Health Organization (WHO). Due to the platform-specific search limitations, we performed simplified keyword searches using the terms ‘artificial intelligence’ and ‘humanitarian’. The Google Scholar and grey literature search were completed on 15 February 2026.

### Study selection

We exported all records retrieved from database searches into EndNote for the initial deduplication and subsequently imported them into Rayyan [19] to further remove any duplicates and perform the literature screening. Two reviewers (MJ, FM) independently screened the titles and abstracts according to the predefined eligibility criteria. They then conducted a blinded full text review of potentially eligible studies; they documented any reasons for exclusion and resolved discrepancies were resolved through discussion and consensus. The records identified through the supplementary Google Scholar and grey literature searches were not imported into Rayyan, but were manually screened against the same predefined eligibility criteria.

### Eligibility criteria

We considered studies if they reported on the application of AI, its development, implementation, or governance within humanitarian contexts. Eligible records included peer-reviewed research articles, conference proceedings, case studies, organisational reports, policy reports, policy briefs, theses, and working papers. Humanitarian contexts included disaster preparedness, response, mitigation, and recovery; AI-supported disaster forecasting and detection; social media analysis for humanitarian response; humanitarian logistics and supply chain management; humanitarian policy analysis; pandemic surveillance; and AI-enabled coordination or transportation systems. We set no geographic or publication date restrictions, but did consider only English language records due to global scope of this review. Given the global scope of the review and its focus on internationally indexed scholarly publications and organisational reports, only English-language records were included. We excluded editorials, commentaries, news articles, opinion pieces, and sources from non-verifiable websites due to them having insufficient methodological transparency.

### Data extraction

Two reviewers (MJ, FM) used a structured data extraction form in Microsoft Excel to record the following information on the included literature: publication characteristics (author, year), geographic focus, humanitarian context, AI applications type, reported use cases, emergency management phase, deployment maturity, evaluation or validation, governance and ethical considerations, participation or local data use, equity-related themes, enabling factors, implementation challenges, study limitations, policy relevance, and stakeholders involved. Discrepancies were resolved through discussion until a consensus was reached.

### Data synthesis

We first synthesised the retrieved evidence using descriptive statistics (*i.e.* frequencies and percentages) according to the included records’ publication characteristics, geographic focus, humanitarian context, AI model family, emergency management phase, deployment maturity, and evaluation. We coded governance, participation, and equity-related themes as non-mutually exclusive categories, since a single record could address more than one theme. We then narratively synthesised the data to identify patterns in implementation, enabling factors, operational challenges, governance considerations, and regional evidence gaps, with a sub-focus on evidence from the MENA region, GCC countries, and Qatar. We did not undertake a formal risk of bias assessment, as this was a scoping review; however, we coded the deployment maturity and evaluation to indicate the stage and strength of the available evidence.

## RESULTS

### Search results

We identified 2,157 records through the database searches, a further 99 from Google Scholar, and 1,412 through targeted grey literature searches across relevant organisational platforms. After deduplication, 1,753 unique records were retained for title and abstract screening, of which 1,603 records were excluded based on predefined eligibility criteria. We assessed the full text of the remaining 150 records and excluded 76; this left 74 records in the final synthesis, which included 57 scholarly publications and 17 grey literature reports (**Figure 1**; Tables S2 and S3 in the **Online Supplementary Document**).

**Figure 1:**
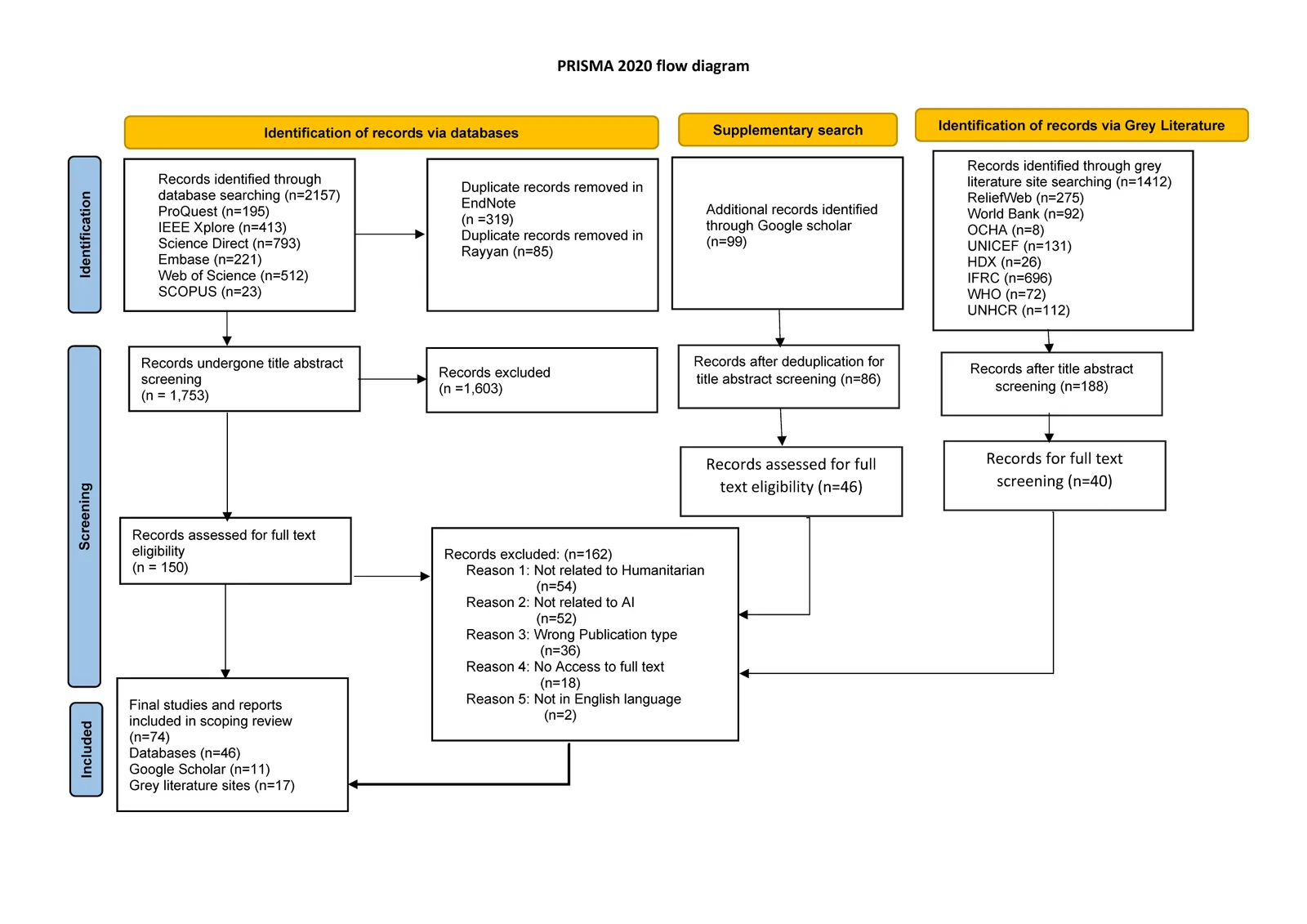
PRISMA 2020 flow diagram showing the identification, screening, eligibility assessment, and inclusion of records in the scoping review.

### Characteristics of included records

The 74 included records were published between 2012 and 2025 (**Table 1**). Among the 57 scholarly publications [5,20–75], we observed a recent rapid growth suggestive of an increase in the use of AI in humanitarian contexts, with only 7 (12.3%) being published before 2021 compared to 38 (66.7%) published between 2023 and 2025. Grey literature reports (n = 17) [76–92] showed a similar trend, with the majority (n = 9, 52.9%) produced in 2024–2025.

**Table 1:** Year-wise distribution of included scholarly publications (n = 57)

Year	n (%)
2016	1 (1.8)
2017	1 (1.8)
2019	1 (1.8)
2020	4 (7.0)
2021	6 (10.5)
2022	6 (10.5)
2023	5 (8.8)
2024	13 (22.8)
2025	20 (35.1)

Geographically, the scholarly publications were predominantly focused on Asia (n = 21, 36.8%), followed by the MENA region (n = 11, 19.3%), multi-country/global contexts (n = 15, 26.3%), North America (n = 7, 12.3%), Europe (n = 2, 3.5%), and sub-Saharan Africa (n = 1, 1.8%). Within the MENA region, evidence included research collaborations linked to Qatar, the United Arab Emirates (UAE), Egypt, Libya, Lebanon, and Kuwait [5,20–75]. Conversely, the grey literature was predominantly global in scope (n = 13, 76.5%) and produced by major multilateral organisations (*e.g.* IFRC, UNICEF, UNHCR, WHO) [76–86,88,91,92]. A smaller number of grey literature reports focused on specific countries or regions, including Ukraine, Pakistan–Afghanistan, and parts of Africa [87,89,90].

### Humanitarian disaster setting and disaster typology

#### Humanitarian context typology

The majority of the included scholarly publications focused on sudden-onset natural disasters (n = 30, 52.6%), including floods, earthquakes, cyclones, landslides, and wildfires that require rapid emergency response and humanitarian assistance [21,24,26,27,38,40,42–44,47,48,52–55,57–60,62,64,72] (**Table 2**). Flood-related disasters represented the most frequent hazard type (n = 11, 19.3%), followed by earthquake-related hazards (n = 8, 14.0%) [21,24,38,40,42,47,52–55,57–60,62,64,72]. Reports on public health emergencies (n = 6, 10.5%) addressed pandemics and disease outbreaks, particularly COVID-19 monitoring, outbreak detection, and public health preparedness [31,34,35,54,65,74]. Conflict and displacement crises were identified in three reports (5.3%) and involved humanitarian context related to armed conflict, forced migration, and refugee displacement, including monitoring of misinformation in conflict settings, infrastructure damage assessment, and displacement analysis in crisis-affected regions such as Gaza and contexts such as the Turkey–Syria earthquake [23,68,71]. In the identified multi-hazard or integrated disaster management systems reports (n = 4, 7.0%), indications of multiple hazard types or broader disaster management were identified [28,30,44,73].

**Table 2:** Typology of humanitarian contexts across included scholarly publications (n = 57)

Humanitarian context typology	Disaster types	n (%)
Natural disasters (sudden-onset hazards)	Floods, earthquakes, cyclones, landslides, wildfires	30 (52.6)
Public health emergencies	Pandemics, disease outbreaks, COVID-19 monitoring	6 (10.5)
Conflict and displacement crises	Armed conflict monitoring, refugee displacement, humanitarian damage assessment	3 (5.3)
Multi-hazard/integrated disaster management	Multiple disaster types or general disaster management	4 (7.0)

Humanitarian contexts were similarly diverse across the grey literature and included conflict and displacement crises, climate-related disasters, public health emergencies, and disaster risk reduction initiatives [76–79,81,83,84,86,90–92]. Examples within the MENA and GCC contexts included conflict-affected settings such as Gaza and Syria, as well as regional initiatives in the UAE [77,78,82,84,85,92].

#### Operational applications of AI in humanitarian setting

AI was applied to a range of operational humanitarian context. Social media-based crisis analytics were one of the most commonly reported applications of AI among the included scholarly publications (n = 13, 22.8%) and were used to support situational awareness, monitor crise, and identify humanitarian needs during emergencies [20,22,25,50,51,56,61,64–68,70]. Several of these systems were developed through international collaborations involving GCC and Qatar-linked research initiatives [67,68,70]. Search-and-rescue (SAR) applications were reported in four studies (7.0%), where AI analysed aerial imagery collected by unmanned aerial vehicles (UAVs) to identify survivors and support rescue operations in disaster-affected areas [5,20,41,65]. Two (3.5%) studies examined humanitarian logistics and supply chain resilience, focusing on AI-supported optimisation of disaster resource allocation and food supply chain management during emergencies [33,36]. In addition, several studies applied AI for disaster monitoring and hazard mapping, including flood detection, wildfire monitoring, and infrastructure damage assessment using satellite imagery and remote sensing data [21,43,52,57–59,62,64,72].

Grey literature reports also described a range of operational uses of AI and digital applications in humanitarian programmes. These included needs assessments, humanitarian reporting and translation, mapping and geospatial analysis, early warning systems (EWS), refugee assistance platforms, and crisis information systems used during disasters, conflicts, and public health emergencies [76,78,83,84,88,90]. In several cases, AI tools were embedded within broader digital platforms designed to support humanitarian coordination, information sharing, and decision-making, rather than focusing on a single analytical model [79,85,87,91].

### AI model families and application patterns

The identified AI models can be broadly categorised into five groups: DL models, classical ML models, hybrid optimisation-based systems, NLP models, and blockchain-integrated systems (**Table 3**). DL approaches were the most reported (n = 27; 47.4%), commonly used to analyse satellite imagery, aerial imagery, and social media data for disaster monitoring and situational awareness [20,43,62,71]. Applications of DL included detection of damaged buildings, wildfire monitoring, and flood mapping from remote sensing imagery [43,62]. Drone-based SAR systems were also described, where AI analysed aerial images to identify survivors [5,41]. Some studies used multimodal models combining text and images for crisis monitoring [20,65]. Within the MENA region, DL was applied in disaster monitoring, earthquake hazard analysis, and satellite-based crisis monitoring, including initiatives involving UAE collaborations and Qatar-linked research institutions such as the Qatar Computing Research Institute [28,40,41,67,68,70]. Classic ML models (n = 17, 29.8%) were frequently applied to hazard risk modelling, geospatial vulnerability assessment, and disaster prediction [40,53,72], while other ML classifiers were used to analyse social media data to identify urgent humanitarian needs during disasters [50,67]. In reports from the MENA, these applications were mainly used in earthquake and flood risk modelling across the Arabian Peninsula and surrounding regions [40,41]. Hybrid systems combining ML with optimisation or decision-support frameworks (n = 8, 14.0%) were primarily used to support humanitarian logistics, resource allocation, and disaster infrastructure management [33,36,44,73]. Furthermore, NLP models (n = 4, 7.0%) were commonly used to analyse social media data during crises supporting tasks such as crisis message classification, extraction of geographic locations from social media posts, and monitoring of migration or displacement patterns [51,61,67,70]. Several NLP-based crisis analytics applications were developed through collaborations involving Qatar-based research initiatives [67,68,70]. Finally, one study (1.8%) used blockchain-integrated AI, where blockchain technology was combined with a drone-based detection system to support secure coordination and data sharing in SAR operations [41].

**Table 3:** AI applications model families identified in included scholarly publications (n = 57)

AI applications model family	n (%)
Deep learning models	27 (47.4)
Machine learning models	17 (29.8)
Hybrid AI systems (ML combined with optimisation or decision-support models)	8 (14.0)
Natural language processing models	4 (7.0)
Blockchain-integrated AI systems	1 (1.8)

AI – artificial intelligence, ML – machine learning

Grey literature described AI being applied more broadly and focused primarily on operational use, rather than technical model development. Reported application of AI included generative AI tools, predictive analytics, geospatial mapping, NLP systems, and digital decision-support platforms used for needs assessments, mapping, humanitarian reporting, refugee assistance, and crisis monitoring [76,77,79,84,86,88–90,92]. Some reports highlighted the use of LLM and generative AI tools in humanitarian workflows [76,77,86], while others focused on ML applications for geospatial mapping and forecasting [83,84,89,90]. Regional examples included Qatar-linked crisis analytics initiatives and UAE-led digital humanitarian coordination platforms [78,84,88].

### Deployment maturity and operational implementation

Retrospective validation using historical or archival data was the most common category related to the deployment maturity of AI (n=28, 37.8%) , followed by 

Retrospective validation using historical or archival data was the most common category, as it was reported in 28 records (37.8%), followed by prototype or experimental models (n = 22, 29.7%) [5,20,21,25,26,28–31,33,35,36,38,40,42,43,47,48,53,58,62,75]. Together, these two categories accounted for 50 of 74 records (67.6%), indicating that most applications of AI were evaluated as retrospective analyses, proof-of-concept models, or experimental systems, rather than as tools embedded in live humanitarian workflows (Table S4 in the **Online Supplementary Document**).

Operational deployment or documented use cases were identified in nine records (12.2%) [67,68,76,83,84,87,88,90,92], while pilot or limited field implementation was reported in three (4.1%) [66,69,89]. Conceptual or proposed frameworks accounted for four records (5.4%) [34,44,73,78], programme, strategy, or guidance documents without a specific AI deployment for six (8.1%) [79–82,85,91], and governance or analytical reports without a specific deployment accounted for two (2.7%) [77,86].

### Evaluation and validation

Retrospective or historical data validation was the most frequently reported form of evidence (n = 28, 37.8%), followed by technical performance or model testing (n = 21, 28.4%) [5,9,20,21,25,26,28–31,35,38,40,42,43,47,48,53,58,62,75]. These records primarily assessed model performance using quantitative indicators such as accuracy, precision, recall, F1-score, area under the curve (AUC), intersection over union (IoU), or related metrics (Table S5 in the **Online Supplementary Document**).

Empirical descriptive or operational evidence was reported on by 12 records (16.2%) [66–68,76,79,84,85,87–90,92]. Less frequent forms of evidence included conceptual or analytical guidance without empirical validation, present in five records (6.8%) [34,73,77,82,86]; programme or strategy reporting without AI-specific validation, present in four records (5.4%) [78,80,81,91]; simulation or scenario-based testing, present in three records (4.1%) [33,36,44]; and tool demonstration with limited user feedback, present in one record (1.4%) [69].

### Emergency management phase coverage

Applications of AI were reported across all the phases of the EMC, including preparedness, mitigation, response, and recovery. Response-phase applications were the most frequently represented, followed by preparedness and mitigation activities, while post-crisis recovery applications were rarely identified (Table S6 in the **Online Supplementary Document**). Preparedness-oriented applications (n = 21, 36.8%) focused on hazard forecasting, EWS, and disaster preparedness planning. Here, AI was applied to predict drought conditions, forecast cyclone trajectories, detect earthquakes, and anticipate disease outbreaks [27,31,32,38,59]. Within the MENA region, preparedness applications included earthquake hazard forecasting and disaster risk modelling studies conducted in Iran and the Arabian Peninsula [25,41,54]. Response-phase applications (n = 27, 47.4%) primarily supported situational awareness, disaster damage assessment, SAR operations, and identification of humanitarian needs during active crises analysing aerial imagery, satellite data, and social media information to detect survivors, assess infrastructure damage, and identify requests for humanitarian assistance [20,26,43,49,63,65,67,71]. Several response-oriented applications were developed through international collaborations involving GCC-linked and Qatar-based research initiatives [33,40,67,68]. Mitigation and prevention applications (n = 13, 22.8%) focused on long-term risk reduction activities such as hazard mapping, vulnerability assessment, and disaster risk planning. Examples included flood risk mapping, landslide susceptibility assessment in refugee settlements, and earthquake vulnerability modelling using geospatial datasets [24,52,58,72]. Some studies also developed decision-support applications to improve resource allocation and disaster logistics planning [33]. Only one study (1.8%) explicitly addressed post-crisis recovery, analysing population mobility and migration dynamics following displacement crises using ML methods applied to social media and migration data [50].

Grey literature reports described AI and digital applications being used across multiple phases of the EMC, particularly for response and preparedness functions [76–78,80,81,84,86–92]. Reported uses included emergency information systems, crisis communication platforms, displacement monitoring, immunisation planning, and EWS [78,83,84,87,89,90,92]. Compared with the scholarly literature, grey literature more often described digital tools that supported multiple stages of humanitarian operations, rather than a single phase of the disaster cycle [76,79,81,84,91].

### Implementation dynamics: governance, enablers, and challenges

Besides describing the contexts and operational uses of AI systems, the included records also described factors influencing their implementation, including governance considerations, enabling conditions, and operational challenges (Table S7 in the **Online Supplementary Document****)**.

#### Governance and ethical considerations

Governance, participation, and equity-related themes were identified across both scholarly publcations and grey literature records (Table S7 in the **Online Supplementary Document**). Protection of vulnerable groups was the most frequently identified governance-related theme, reported on by 21 records (28.4%), followed by privacy and data protection, present in 16 records (21.6%); digital inclusion or access, present in 10 records (13.5%); cybersecurity or secure data sharing, present eight records (10.8%); and human oversight or human-in-the-loop approaches, present in seven records (9.5%). Other themes were less frequently reported on, including transparency or explainability, bias or fairness, legal or policy frameworks, accountability, information integrity or disinformation, and data sovereignty or local ownership.

Explicit discussion was limited of formal governance frameworks for responsible AI deployment across the included scholarly publications. When governance issues were addressed, they were usually linked to specific technical or operational risks, rather than comprehensive regulatory or ethical frameworks. For example, blockchain-integrated SAR systems highlighted secure data sharing between response teams [40], while refugee-support chatbot systems emphasised privacy-preserving design and transparency regarding the use of AI tools [66]. Cybersecurity considerations were also reported on in the context of AI-enabled communication network systems for disaster response [73]. Some hazard modelling studies used explainable AI techniques to improve the interpretability of predictions for emergency planners and decision-makers [41]. Within the MENA and GCC evidence base, governance considerations were mainly discussed in relation to data security, model transparency, and responsible use of digital systems in crisis settings, with limited reporting of broader regulatory or ethical governance frameworks.

Governance issues were discussed more explicitly in the grey literature (Table S7 in the **Online Supplementary Document**), which frequently addressed data protection, privacy, transparency, bias, accountability, cybersecurity, and responsible AI use in humanitarian contexts [76,77,79,83,84,86–88]. Several reports indicated a gap between the expanding use of AI applications and the limited availability of formal organisational policies, staff guidance, or governance frameworks to regulate their deployment [76,86]. Reports focusing on refugee assistance, child health programmes, and humanitarian information systems also emphasised responsible data use and the protection of vulnerable populations [83,84,87]. Concerns about information integrity and AI-enabled disinformation were particularly prominent in conflict settings [77]. In reports with MENA or GCC relevance, governance discussions were mainly related to secure digital coordination platforms and responsible digital systems in crisis settings, including UAE digital response platforms and regional conflict monitoring initiatives [77,78].

Participation-related findings showed a distinction between the use of local or crisis-specific data and the direct involvement of affected populations. Local or secondary data were used in 20 records (27.0%) [22,37,45,49,50,55,56,59,60,63,65,70,71,74,76,77,83,84,90,92], but affected-community participation or co-design was reported on by only eight records (10.8%) [66,79–81,85,87,90,92], while practitioner, volunteer, or human-in-the-loop involvement were dealt with by seven records (9.5%) [5,29,67,69,76,81,88].

Equity-related population themes were also unevenly represented. Refugees, migrants, displaced populations, or legal status were mentioned in nine records (12.2%) [50,66,68,72,74,79,85,90,91], digital divide or access-related concerns in six (8.1%) [73,76,83,85,87,91], conflict or war-affected populations in five (6.8%) [22,71,77,82,87], children in two (2.7%) [83,84], and gender in one (1.4%) [66].

#### Enabling factors for adoption

Across the scholarly publications, enabling factors for AI adoption were mainly related to data availability, digital infrastructure, methodological advances, and institutional collaboration. Public disaster datasets, satellite imagery archives, geospatial data, and social media streams supported applications in disaster monitoring, forecasting, situational awareness, and humanitarian needs identification [22,25,32,34,50,52,57,67,71]. Pre-trained models, transfer learning, and explainable AI approaches were also used to improve performance in data-constrained settings and support interpretation by emergency planners [5,41,59,65]. Collaboration between universities, research institutes, humanitarian organisations, and international agencies facilitated data sharing and operational use of AI tools, including MENA- and GCC-linked initiatives involving satellite data, crisis monitoring, and disaster risk assessment [22,23,41,66,68,71].

Across the grey literature, enabling factors were most often linked to digital access, organisational commitment to innovation, cross-sector partnerships, and the availability of AI-enabled tools, satellite imagery, and geospatial platforms [76,78,79,81,83,84,86,88]. Partnerships involving humanitarian organisations, UN agencies, governments, research institutions, and technology providers were repeatedly identified as drivers of digital innovation, while community engagement, local adaptation, and human oversight were described as important conditions for responsible AI use in humanitarian settings [79,83–86,88].

#### Implementation challenges and operational barriers

Among the several challenges affecting the implementation of AI applications in humanitarian setting, the most commonly discussed issues were related to data availability and quality, including limited labelled data sets, incomplete satellite imagery coverage, and restricted access to geolocated social media data during rapidly evolving emergencies [50,57,58,61,62,68,71,74]. These limitations were evident during the early stages of disasters, when reliable information is scarce and data collection systems may be disrupted [25,50,64,67]. Application-specific challenges, including difficulties analysing informal and multilingual social media data [50,51,61,64,68,70] and environmental constraints affecting satellite-based monitoring, such as cloud cover and irregular imagery availability [57–59,71] were also reported on by MENA and GCC-related studies, particularly regarding limited labelled data sets and difficulties validating AI outputs in conflict or disaster contexts [41,68,71].

Across the grey literature, implementation challenges were most associated with governance gaps, limited organisational readiness, and barriers to digital access [76,77,79,84,86,88]. Although AI applications are increasingly used in humanitarian programmes, institutional policies, staff training, and governance frameworks often remain underdeveloped [76,87]. Reports also emphasised the importance of digital inclusion and community-centred design, particularly in migration and displacement programmes, where vulnerable populations may face barriers to accessing digital services [79,85]. Additional challenges included misinformation, information verification difficulties, and risks associated with digital information systems in conflict settings [77,87,90].

## DISCUSSION

In this scoping review, we mapped the evidence on applications of AI in humanitarian contexts, synthesising 74 records from scholarly publications and grey literature. The findings show a rapidly evolving but uneven evidence base, with a concentration of applications in specific disaster types and a persistent gap between technological development and operational implementation.

### Growth of AI evidence, but uneven regional distribution

The recent growth of AI-related evidence reflects wider advances in digital technologies, machine learning methods, remote sensing, and humanitarian data systems. Similar trends have been reported in previous reviews of AI in disaster management and humanitarian response [6,13,93]. However, the evidence remains geographically uneven: although we identified some MENA- and GCC-linked studies, this evidence base was limited compared with the ones originating from Asia and global multi-country initiatives. This is important because MENA countries face overlapping humanitarian challenges, including conflict, displacement, climate-related hazards, and public health emergencies, while also investing in AI and digital transformation strategies [15,16]. More regional studies should assess whether AI tools suit local priorities, languages, governance structures, and operational realities.

### AI use is concentrated in response and sudden-onset disasters

AI applications were concentrated mainly in sudden-onset disasters and in preparedness or response activities. This pattern reflects the availability of satellite imagery, social media data, hazard datasets, and other digital sources that can be used for prediction, monitoring, and rapid situational awareness. Previous studies have similarly identified remote sensing, predictive analytics, and crisis informatics as prominent areas of AI use in disaster management [6,94–96]. However, this concentration also shows that humanitarian AI remains more developed for acute crisis detection than for longer-term humanitarian needs. Recovery, protracted displacement, conflict settings, public health emergencies, and humanitarian logistics received less attention. This gap is important because many humanitarian crises are prolonged and require sustained support beyond the immediate response phase. Future research should examine how AI can support recovery, resilience, service continuity, and long-term planning across the full EMC [6,11].

### The implementation gap between innovation and practice

A central finding of our review is the gap between AI development and operational use, with most applications being prototypes, experimental models, or retrospectively validated tools, and with fewer records documented field implementation, operational deployment, or institutional integration. Evaluation was also largely technical, with limited prospective assessment of usability, effectiveness, decision-making impact, or outcomes in real humanitarian settings.

Current literature largely demonstrates technical feasibility, rather than operational value. Similar concerns have been raised in previous work on the difficulty of translating AI innovations into humanitarian practice [8,97]. The barriers we identified here, including data limitations, infrastructure constraints, fragmented information systems, workforce capacity gaps, and governance concerns, help explain why technically promising tools may not easily become usable humanitarian systems. Simultaneously, public datasets, satellite imagery archives, explainable AI approaches, and cross-sector partnerships were important enablers. This suggests that future works should place greater emphasis on implementation science, operational evaluation, and user-centred validation rather than focusing mainly on model development.

### Governance, participation, and equity remain underdeveloped

Governance, participation, and equity considerations were reported on inconsistently in literature included in this review. Scholarly studies tended to focus on algorithms, model performance, and technical outputs, while grey literature more often discussed privacy, data protection, accountability, transparency, cybersecurity, digital inclusion, and protection of vulnerable populations. This separation between technical evidence and governance evidence has also been noted in the wider humanitarian AI literature [6,7,10,98].

Affected community participation was also limited. Although many records used local or crisis-specific datasets, fewer described direct involvement of affected populations in the design, validation, or governance of AI systems. This distinction matters because using local data is not the same as ensuring local participation or accountability. Participatory and community-centred approaches are important for improving trust, contextual relevance, and responsible adoption in humanitarian settings [7,10,98].

Equity-related reporting was similarly limited in scope, with some records addressing refugees, migrants, displaced populations, legal status, conflict-affected communities, children, or gender, but broader dimensions such as disability, ethnicity, and algorithmic fairness being rarely examined. Few records also discussed how international humanitarian law, refugee law, AI regulatory frameworks, or accountability mechanisms would apply if AI-assisted decisions caused harm. These gaps suggest that responsible AI principles have not yet been fully integrated into humanitarian AI development and implementation.

### Implications for future research and practice

Our findings suggest that future humanitarian AI research should move beyond technical development toward implementation science, operational evaluation, and governance research. Greater emphasis should be placed on prospective field studies, user-centred validation, participatory design approaches, and equity-focused assessments. Researchers should also prioritise applications supporting recovery, resilience, protracted crises, displacement settings, and public health emergencies, which remain comparatively underexplored. Policymakers and humanitarian organisations should embed governance, accountability, and community participation from the outset. Multidisciplinary collaboration is also needed to align AI innovation with humanitarian values and operational realities.

## CONCLUSIONS

In this scoping review, we found that humanitarian AI applications primarily support disaster monitoring, prediction, situational awareness, crisis informatics, SAR, and coordination. Evidence was concentrated on sudden-onset disasters and preparedness or response, with less attention given to recovery, protracted crises, displacement, public health emergencies, humanitarian logistics, and the MENA and GCC regions. Most applications were prototypes or retrospectively validated tools, with limited field implementation or institutional integration. Governance, participation, equity, and accountability were also inconsistently addressed. Future research should prioritise real-world evaluation, community participation, governance, and context-specific implementation to ensure that AI meaningfully supports humanitarian decision-making.

## Additional material


Online Supplementary Document

## Data Availability

**Data availability:** The data supporting the findings of this study are available within the article.
