# Supplementary material for: From innovation to implementation: a global scoping review of artificial intelligence applications, governance, and challenges in humanitarian contexts
Source: J Glob Health. 2026 Oct 19;16:04359. doi: 10.7189/jogh.16.04359 (PMC13679343; doi:10.7189/jogh.16.04359)
Supplement: Supplementary file 1 [file jogh-16-04359-s001.pdf]

**Supplement to: Jose M, Mueen F, Qadir J, Abd-alrazaq A, Kwalombota K, Maddah D. From innovation to implementation: a global scoping review of artificial intelligence applications, governance, and challenges in humanitarian contexts. J Glob Health. 2026;16:04359.**

**Table S1.** Deployment maturity of AI applications across included records (n = 74)

| Deployment maturity category | Scholarly articles, n | Grey literature, n | Total, n | Percentage (%) |
|---|---|---|---|---|
| Retrospective validation using historical/archival data | 28 | 0 | 28 | 37.8 |
| Prototype / experimental model | 22 | 0 | 22 | 29.7 |
| Operational deployment/use case documented | 2 | 7 | 9 | 12.2 |
| Programme/strategy/guidance without specific AI deployment | 0 | 6 | 6 | 8.1 |
| Conceptual / proposed framework | 3 | 1 | 4 | 5.4 |
| Pilot / limited field implementation | 2 | 1 | 3 | 4.1 |
| Governance/analytical report without specific deployment | 0 | 2 | 2 | 2.7 |

**Table S2.** Type of evaluation/validation evidence reported across included records (n = 74)

| Evidence type | Scholarly articles, n | Grey literature, n | Total, n | Percentage (%) |
|---|---|---|---|---|
| Retrospective / historical data validation | 28 | 0 | 28 | 37.8 |
| Technical performance / model testing | 20 | 1 | 21 | 28.4 |
| Empirical descriptive / operational evidence | 3 | 9 | 12 | 16.2 |
| Conceptual/analytical guidance; no empirical validation | 2 | 3 | 5 | 6.8 |
| Programme/strategy reporting without AI-specific validation | 0 | 4 | 4 | 5.4 |
| Simulation / scenario-based testing | 3 | 0 | 3 | 4.1 |
| Tool demonstration / limited user feedback | 1 | 0 | 1 | 1.4 |

**Table S3.** Emergency management phase coverage across Scholarly articles (n = 57)

| **Emergency management phase** | **Number of studies (n)** | **Percentage (%)** |
|---|---|---|
| Preparedness / early warning / prediction | 21 | 36.8 |
| Mitigation / prevention | 13 | 22.8 |
| Response | 27 | 47.4 |
| Post-crisis / recovery | 1 | 1.8 |

**Table S4.** Governance, participation, and equity-related themes reported across included records (n = 74)

| **Theme / flag** | **Scholarly articles, n** | **Grey literature, n** | **Total, n** | **Percentage (%)** |
|---|---|---|---|---|
| **Governance-related themes** | | | | |
| Protection / vulnerable groups | 8 | 13 | 21 | 28.4 |
| Privacy / data protection | 7 | 9 | 16 | 21.6 |
| Digital inclusion / access | 2 | 8 | 10 | 13.5 |
| Cybersecurity / secure data sharing | 5 | 3 | 8 | 10.8 |
| Human oversight / human-in-the-loop | 4 | 3 | 7 | 9.5 |
| Bias / fairness | 2 | 3 | 5 | 6.8 |
| Transparency / explainability | 4 | 1 | 5 | 6.8 |
| Legal / policy / regulatory framework | 0 | 5 | 5 | 6.8 |
| Accountability | 1 | 3 | 4 | 5.4 |
| Information integrity / disinformation | 1 | 3 | 4 | 5.4 |
| Data sovereignty / local ownership | 0 | 1 | 1 | 1.4 |
| **Participation and local-data themes** | | | | |
| Local / secondary data used | 14 | 6 | 20 | 27.0 |
| Affected-community participation / co-design | 1 | 7 | 8 | 10.8 |
| Practitioner / volunteer / human-in-the-loop involvement | 4 | 3 | 7 | 9.5 |
| **Equity-related population themes** | | | | |
| Refugees / migrants / displaced populations / legal status | 5 | 4 | 9 | 12.2 |
| Digital divide / access | 1 | 5 | 6 | 8.1 |

| | | | | |
|---|---|---|---|---|
| Conflict / war-affected populations | 2 | 3 | 5 | 6.8 |
| Children | 0 | 2 | 2 | 2.7 |
| Gender | 1 | 0 | 1 | 1.4 |

**Table S5:** Population-Concept-Context

| Population: Humanitarian | Concept: Artificial Intelligence | Context: |
|---|---|---|
| “humanitarian care” OR “humanitarian assistance” OR humanitarian OR humanitari* OR “emergency management” OR emergency OR “crisis management” OR “non-crisis management” OR “disease prevention” OR “disaster management” OR “resource allocation” OR “humanitarian relief” OR relief OR “international corporation” OR “crisis response” OR war OR conflict OR “food relief” OR “humanitarian operation” OR “humanitarian organizations” | "Artificial intelligence" OR AI “machine learning” OR “deep learning” OR ML OR “intelligent robots” OR “learning systems” OR “satellite imagery” OR “chatbots” OR “computer vision” OR “predictive analytics” | MENA OR “Middle east” OR “North Africa” OR Gulf OR “Gulf region” OR GCC OR “Arab world” |

**Table S6:** Characteristics of included scholarly articles (n = 57)

| Sl No. | Ref No. | Author(s) | Title | Year | Geographic focus | AI application / model family | Emergency phase | Humanitarian context | Deployment maturity | Evaluation / validation evidence |
|---|---|---|---|---|---|---|---|---|---|---|
| 1 | 18 | Ahmad A. Bany Abdelnabi, Bulent Soykan, Ghaith Rabadi | Deep Learning for Human Detection from Aerial Images in Search and Rescue Missions | 2024 | USA | Deep learning (computer vision) | Response | Search-and-rescue operations | Prototype / experimental model | Technical performance / model testing |
| 2 | 19 | Abdullah Alsalmani, Mohamed Abduljawad, Mohammed Osama Elnour | Towards a Smart Low-cost Deluge Forecasting System | 2023 | UAE | Machine learning | Preparedness | Flood early warning systems | Prototype / experimental model | Technical performance / model testing |
| 3 | 20 | Fatima K. Abu Salem, Roaa Al Feel, Shady Elbassuoni, Hiyam Ghannam, Mohamad Jaber, May Farah | Meta-learning for fake news detection surrounding the syrian war | 2021 | Lebanon | Machine learning | Preparedness | Conflict misinformation monitoring | Retrospective validation using historical/archival data | Retrospective / historical data validation |
| 4 | 21 | Junaid Ahmad, Irfan Ahmad, Qing Miao, Zhaohui Su | Forecasting the human cost of disasters under Sustainable Development Goal: A time series analysis using Facebook Prophet model | 2025 | China | Machine learning | Preparedness | Disaster risk prediction | Retrospective validation using historical/archival data | Retrospective / historical data validation |
| 5 | 22 | Mohsen Ahmadi Mahyat Shafapourtehran, Haluk Ozener , | Eigenvector spatial filtering enhancing natural hazards vulnerability | 2024 | Turkey / Iran / Qatar | Machine learning | Preparedness / Mitigation | Seismic hazard mapping | Retrospective validation using historical/archival data | Retrospective / historical data validation |

| | | | | | | | | | | |
|---|---|---|---|---|---|---|---|---|---|---|
| | | Orkut Yilmaz ,Bahareh Kalantar, Farzin Shabani | assessment in a susceptible urban environment: A case study of Izmir earthquake in Turkey | | | | | | | |
| 6 | 23 | Hadi Akbarian ,Mohammad Gheibi, Mostafa Hajiaghaei-Keshteli ,Mojtaba Rahmani | A hybrid novel framework for flood disaster risk control in developing countries based on smart prediction systems and prioritized scenarios | 2022 | Iran | Machine learning and neural networks | Preparedness / Response / Mitigation | Flood disaster management | Prototype / experimental model | Technical performance / model testing |
| 7 | 24 | Sarah Al-Shareeda, Yasar Celik, Bilge Bilgili, Ahmed Al-Dubai, and Berk Canberk | Accurate AI-Driven Emergency Vehicle Location Tracking in Healthcare ITS's Digital Twin | 2025 | Turkey | Machine learning and deep learning | Preparedness / Response | Emergency health transport systems | Prototype / experimental model | Technical performance / model testing |
| 8 | 25 | Amobichukwu C. Amanambu , Joann Mossa and Yin-Hsuen Chen | Hydrological Drought Forecasting Using a Deep Transformer Model | 2022 | USA | Deep learning | Preparedness | Climate-related disaster prediction | Retrospective validation using historical/archival data | Retrospective / historical data validation |
| 9 | 26 | Branislava Jankovic , Sabina Jangirova , Waseem Ullah , Latif U. Khan ,and Mohsen Guizan | Disaster Detection on the Fly: Optimized Transformers for uavs | 2025 | UAE | Deep learning | Response | Aerial disaster monitoring | Prototype / experimental model | Technical performance / model testing |
| 10 | 27 | Mehdi Ben Lazreg, Nadia Noori, Tina Comes, Morten Goodwin | Not a Target. A Deep Learning Approach for a Warning and Decision Support System to Improve Safety and Security of Humanitarian Aid Workers | 2019 | Norway | Deep learning | Response | Aid worker safety monitoring | Prototype / experimental model | Technical performance / model testing |

| | | | | | | | | | | |
|---|---|---|---|---|---|---|---|---|---|---|
| 11 | 28 | Chengyi Qu, Xiaodong Hao, Xu Huang, Wenbin Guo, Jizhuang Hui, Hong Wang | Cross-domain adversarial structural deformation model for post-disaster image generation | 2025 | China | Deep learning | Response | Disaster damage assessment | Prototype / experimental model | Technical performance / model testing |
| 12 | 29 | Cenk Serhan Ozverel ,Abdullahi Garba Usman, Nazife Sultanoglu, Berna Uzun, Cemile Bagkur, Dilber Uzun Ozsahin,Tamer Sanlidag | Implementation of Artificial Intelligence-Based Models with Improved Hybrid Techniques in Forecasting Possible Future Outbreaks: Which Agents and When? | 2024 | Turkey | Hybrid machine learning models | Preparedness | Public health emergency forecasting | Prototype / experimental model | Technical performance / model testing |
| 13 | 30 | C. Purna Chand, M.M. Ali, Borra Himasri,Mark A. Bourassa, Yangxing Zheng | Predicting Indian Ocean Cyclone Parameters Using an Artificial Intelligence Technique | 2022 | India | Artificial neural networks | Preparedness | Cyclone early warning | Retrospective validation using historical/archival data | Retrospective / historical data validation |
| 14 | 31 | Mengfei Chen, Mohamed Kharbeche, Mohamed Haouari,Weihong Guo (Grace) | A Markov decision process model for enhancing resilience in food supply chains during natural disasters | 2025 | USA / Qatar | Optimization-based AI models | Response | Humanitarian logistics | Prototype / experimental model | Simulation / scenario-based testing |
| 15 | 32 | Alfredo Cuzzocrea, Carson K. Leung, Siyuan Shang, Yan Wen | A Big Data Management and Analytics Framework for Supporting Machine Learning, OLAP, and Visualization on Big COVID-19 Data | 2025 | USA / Canada / Italy | Machine learning | Preparedness | Pandemic data analysis | Conceptual / proposed framework | Conceptual / analytical guidance; no empirical validation |
| 16 | 33 | Dicky Dwi Putra, Mohammad | Design of Smart-Gate Based on Artificial Intelligence Possibly | 2021 | Indonesia | Machine learning | Mitigation | Pandemic prevention measures | Prototype / experimental model | Technical performance / model testing |

| | | | | | | | | | | |
|---|---|---|---|---|---|---|---|---|---|---|
| | | Febriyanto, Muhammad Miqdad Nadra, Wervyan Shalannanda,Elsa Ramadhani Firzal, Achmad Munir | for COVID-19 Early Prevention at Public Area | | | | | | | |
| 17 | 34 | Kassem Danach , Hassan Harb , Louai Saker and Ali Raad | Quantum-Inspired Hyperheuristic Framework for Solving Dynamic Multi-Objective Combinatorial Problems in Disaster Logistics | 2025 | Lebanon / Kuwait | Optimization-based AI models | Response | Relief resource allocation | Prototype / experimental model | Simulation / scenario-based testing |
| 18 | 35 | Ghadah Adel, Yuping Wang | Detecting and Classifying Humanitarian Crisis in Arabic Tweets | 2020 | China | Machine learning and NLP | Response | Crisis informatics | Retrospective validation using historical/archival data | Retrospective / historical data validation |
| 19 | 36 | Hasan Awad, Fehmida Usmani, Emanuele Virgillito, Rudi Bratovich, Stefano Straullu, Francesco Aquilino, Roberto Proietti, Rosanna Pastorelli and Vittorio Curri | Artificial Intelligence Driven Earthquakes Early Detection in Noisy Urban Areas | 2024 | Turkey / Pakistan | Deep learning | Preparedness | Seismic early warning | Prototype / experimental model | Technical performance / model testing |
| 20 | 37 | Iqra Atif ,Frederick Thomas Cawood, Muhammad Ahsan | An Integrated Approach of GIS and Machine Learning to Assess the Spatio-Temporal Earthquake | 2021 | South Africa | Machine learning with geospatial analysis | Preparedness / Mitigation | Seismic risk mapping | Retrospective validation using historical/archival data | Retrospective / historical data validation |

| | | | | | | | | | | |
|---|---|---|---|---|---|---|---|---|---|---|
| | | Mahboob, Sarfraz Ali | Vulnerability in South Africa | | | | | | | |
| 21 | 38 | Iman Mohamad Chaabi, Shahmir Khan Mohammed, Maha Kadadha, Rabeb Mizouni, Shakti Singh, Hadi Otrok | Leveraging Blockchain and Deep Learning for UAV-assisted Search and Rescue Missions | 2024 | UAE | Deep learning and blockchain integration | Response | Search-and-rescue operations | Prototype / experimental model | Technical performance / model testing |
| 22 | 39 | Ratiranjan Jena,Abdallah Shanableha, Rami Al-Ruzouq, Biswajeet Pradhan, Mohamed Barakat A. Gibril , Mohamad Ali Khalil , Omid Ghorbanzadehe, Pedram Ghamisi | Earthquake spatial probability and hazard estimation using various explainable AI (XAI) models at the Arabian peninsula | 2023 | UAE / Australia / Germany | Machine learning | Preparedness / Mitigation | Seismic hazard mapping | Retrospective validation using historical/archival data | Retrospective / historical data validation |
| 23 | 40 | Kibitok Abraham, Moataz Abdelwahab and Mohammed Abo-Zahhad | Image Classification of Natural Disasters Using Different Deep Learning Models | 2022 | Egypt | Deep learning | Response | Disaster monitoring | Prototype / experimental model | Technical performance / model testing |
| 24 | 41 | Nema Ahmed, Somaya Al-Maadeed | Enhancing Post-Disaster Survivor Detection Using UAV Imagery and Transfer Learning Strategies | 2024 | MENA region | Deep learning | Response | Search-and-rescue operations | Prototype / experimental model | Technical performance / model testing |
| 25 | 5 | Murad Al-Rajab, Mahmoud Ahmed Soliman, Mohammed | Artificial Intelligence for Real-Time Disaster Management: A New | 2024 | Global | Machine learning and reinforcement learning | Response | Emergency coordination | Prototype / experimental model | Technical performance / model testing |

| | | | | | | | | | | |
|---|---|---|---|---|---|---|---|---|---|---|
| | | Nezam Al'asad, Yaqoob Arshad Jamil, Samia Loucif, Ibrahim Al Qatawneh | Platform for Efficient Recovery and Volunteer Training | | | | | | | |
| 26 | 42 | Mehmet Basaran, Semih Aktas, Buse Bilgin | 6G Network Slicing Vision for Post-Disaster: AI-Enabled User Prioritization and Energy Management | 2024 | Turkey | AI-enabled communication optimization | Response / Mitigation | Disaster communication networks | Conceptual / proposed framework | Simulation / scenario-based testing |
| 27 | 43 | Marwen Bouabid and Mohamed Farah | DETECTING LOCAL CRISIS EVENTS: A CASE STUDY ON THE COLORADO WILDFIRES THROUGH SOCIAL MEDIA AND SATELLITE IMAGERY | 2024 | USA | Machine learning and deep learning | Response | Wildfire monitoring | Retrospective validation using historical/archival data | Retrospective / historical data validation |
| 28 | 44 | Lina Bouchelkia, Ahmed Tahraoui, Radja Kheddam | POST-FLOOD DISASTER MAPPING USING DEEP NEURAL NETWORK | 2024 | Libya | Deep learning | Response / Mitigation | Flood disaster mapping | Retrospective validation using historical/archival data | Retrospective / historical data validation |
| 29 | 45 | Athakorn KENGPOL and Chawantorn CHANCHITTAKARN | The Design of Machine Learning for Searching Casualty People in the Gulf of Thailand | 2023 | Thailand | Deep learning | Preparedness / Response | Maritime search-and-rescue | Prototype / experimental model | Technical performance / model testing |
| 30 | 46 | Shahnawaz Khan, Bharavi Mishra, Ashraf Ali, Mustafa Kamal, Mohammed Redha Qader, | Face Mask Detection from Live-Stream Surveillance Video using Convolutional Neural Network | 2021 | Global | Deep learning | Mitigation | COVID-19 mitigation | Prototype / experimental model | Technical performance / model testing |

| | | | | | | | | | |
|---|---|---|---|---|---|---|---|---|---|
| | | Mohammad Haider | | | | | | | |
| 31 | 47 | Rabindra Lamsal, T. V. Vijay Kumar | Twitter-Based Disaster Response Using Machine Learning | 2023 | Nepal / India | Machine learning | Response | Crisis informatics | Retrospective validation using historical/archival data | Retrospective / historical data validation |
| 32 | 48 | Richard Lemoine-Rodríguez , Johannes Mast, Martin Mühlbauer, Nico Mandery, Carolin Biewer, Hannes Taubenb¨ock | The voices of the displaced: Mobility and Twitter conversations of migrants of Ukraine in 2022 | 2024 | Ukraine crisis | Deep learning | Post-crisis / Recovery | Migration analytics | Retrospective validation using historical/archival data | Retrospective / historical data validation |
| 33 | 49 | Yixuan Liu Alim Samat , Peijun Du, Jin Chen, Jilili Abuduwaili, Kaiyue Luo, Enzhao Zhu, Dana Shokparova | High-resolution spatiotemporal analysis and driver attribution of floods in Kazakhstan using SHAP and remote sensing integration | 2025 | Kazakhstan | Machine learning with explainable AI | Mitigation / Prevention | Flood risk monitoring | Retrospective validation using historical/archival data | Retrospective / historical data validation |
| 34 | 50 | Surfiarti Maharani, Ashar Muda Lubis, Elfi Yuliza, Rida Samdara, Bambang Setiadi | Landslide susceptibility mapping along Taba Penanjung area, Bengkulu province, Indonesia using machine learning (ML) techniques | 2025 | Indonesia | Machine learning | Mitigation | Landslide risk assessment | Retrospective validation using historical/archival data | Retrospective / historical data validation |
| 35 | 51 | Aaisha Makkar, Myra Conway, Farid Meziane, Vinaytosh Mishra, | A Multi-Modal Bio-Medical Data Analysis Framework: A case study for Pandemic Preparedness | 2024 | Global | Deep learning with explainable AI | Mitigation | Pandemic preparedness | Prototype / experimental model | Technical performance / model testing |

| | | | | | | | | | | |
|---|---|---|---|---|---|---|---|---|---|---|
| | | Ashutosh Kumar Singh | | | | | | | | |
| 36 | 52 | Salma Ommi , Mohammad Hashemi | Machine learning technique in the north zagros earthquake prediction | 2024 | Iran | Machine learning | Preparedness | Seismic forecasting | Retrospective validation using historical/archival data | Retrospective / historical data validation |
| 37 | 53 | Pradeep Kumar Roy , Abhinav Kumar, Jyoti Prakash Singh , Yogesh Kumar Dwivedi , Nripendra Pratap Rana , Ramakrishnan Raman | Disaster related social media content processing for sustainable cities | 2021 | USA / India | Deep learning | Response | Crisis social media analytics | Retrospective validation using historical/archival data | Retrospective / historical data validation |
| 38 | 54 | Rizwan Sadiq, Zainab Akhtar, Muhammad Imran, Ferda Ofli | Integrating remote sensing and social sensing for flood mapping | 2022 | Somalia / India / Italy / Bahamas | Deep learning (remote sensing) | Mitigation | Flood mapping | Retrospective validation using historical/archival data | Retrospective / historical data validation |
| 39 | 55 | Ira Karrel San Jose, Sesa Wiguna, Ryohei Kametaka, Bruno Adriano, Erick Mas, Shunichi Koshimura | Evaluation of open-source SAR-based flood datasets for flood extent mapping in emergency settings | 2025 | Philippines / Nigeria / Japan | Deep learning (satellite imagery) | Mitigation | Flood extent mapping | Retrospective validation using historical/archival data | Retrospective / historical data validation |
| 40 | 56 | MUHAMMAD TARIQ SHAHEEN, HAFSA IQBAL, NUMAN KHURSHID, HALEEMA SADIA, NASIR SAEED | Swinsegformer: Advancing Aerial Image Semantic Segmentation for Flood Detection | 2025 | USA / Pakistan | Deep learning (transformer-based) | Mitigation | Flood monitoring | Prototype / experimental model | Technical performance / model testing |

| | | | | | | | | | | |
|---|---|---|---|---|---|---|---|---|---|---|
| 41 | 57 | Yuran Sun, Shih Kai Huang, Xilei Zhao | Predicting Hurricane Evacuation Decisions with Interpretable Machine | 2024 | USA | Machine learning | Preparedness | Hurricane evacuation planning | Retrospective validation using historical/archival data | Retrospective / historical data validation |
| 42 | 58 | Reem Suwaileh , Tamer Elsayed , Muhammad Imran , Hassan Sajjad | When a disaster happens, we are ready: Location mention recognition from crisis tweets | 2022 | Global | Natural language processing (NLP) | Response | Crisis geolocation extraction | Retrospective validation using historical/archival data | Retrospective / historical data validation |
| 43 | 59 | Burak Tasci, Madhav R. Acharya, Mehmet Baygin, Sengul Dogan, Turker Tuncer, Samir Brahim Belhaouari | Incr: Inception and concatenation residual block-based deep learning network for damaged building detection using remote sensing images | 2023 | Turkey | Deep learning | Response | Post-disaster damage assessment | Retrospective validation using historical/archival data | Retrospective / historical data validation |
| 44 | 60 | Kathiravan Thangavel, Dario Spiller, Roberto Sabatini, Pier Marzocca, Marco Esposito | Near Real-Time Wildfire Management Using Distributed Satellite System | 2022 | Australia | Deep learning | Mitigation | Wildfire detection | Prototype / experimental model | Technical performance / model testing |
| 45 | 61 | Irfan Ullah, Sharifullah Khan, Muhammad Imran, Young-Koo Lee | Rweetminer: Automatic identification and categorization of help requests on twitter during disasters | 2021 | Global | Machine learning | Response | Humanitarian aid coordination | Retrospective validation using historical/archival data | Retrospective / historical data validation |
| 46 | 62 | MOHAMED WAHBA, EMAD MABROUK, YOUSSEF M. YOUSSEF , MOHAMED SABER , NASSIR | Enhanced Hazard Mapping for Flood and Landslide Risks Using Memetic Programming and Machine Learning Techniques to Support Sustainable Development Goals | 2025 | Japan | Machine learning | Preparedness | Disaster risk mapping | Retrospective validation using historical/archival data | Retrospective / historical data validation |

| | | | | | | | | | | |
|---|---|---|---|---|---|---|---|---|---|---|
| | | ALARIFI, NAZIH Y. REBOUH , AND MAHMOUD M. MANSOUR | | | | | | | | |
| 47 | 63 | Ferda Ofli, Firoj Alam, Muhammad Imran | Analysis of Social Media Data using Multimodal Deep Learning for Disaster Response | 2020 | USA / Mexico | Multimodal deep learning | Response | Situational awareness | Retrospective validation using historical/archival data | Retrospective / historical data validation |
| 48 | 64 | Maxwell Gerry; Olivia Kochol; Delia Mahoney; Devin Mihaichuk; Matt Walsh | Providing Chatbot Assistance for Ukrainian Refugee Women in Romania | 2025 | Romania | Natural language processing (chatbot) | Response | Refugee support services | Pilot / limited field implementation | Empirical descriptive / operational evidence |
| 49 | 65 | Muhammad Imran, Firoj Alam, Ferda Ofli, Micheal Aupetit | Enabling Rapid Disaster Response Using Artificial Intelligence and Social Media | 2017 | Global | Machine learning | Response | Crisis informatics | Operational deployment / documented use case | Empirical descriptive / operational evidence |
| 50 | 66 | NOORA AL-EMADI, MUHAMMAD IMRAN, YIN YANG, INGMAR WEBER, FABJAN LASHI, GAIA RIGODANZA, IVANA HAJŽMANOVÁ, AND FERDA OFLI | AI-Driven Disaster Response and Displacement Monitoring | 2025 | Turkey / Syria | Natural language processing | Response | Conflict displacement analytics | Operational deployment / documented use case | Empirical descriptive / operational evidence |
| 51 | 67 | Tomaz Logar, Joseph Bullock, Edoardo Nemni, | Pulsesatellite: A Tool Using Human-AI Feedback Loops for | 2020 | Bangladesh / Somalia | Human-AI system with | Response | Refugee camp mapping | Pilot / limited field implementation | Tool demonstration / limited user feedback |

| | | | | | | | | | | |
|---|---|---|---|---|---|---|---|---|---|---|
| | | Lars Bromley, John A. Quinn,Miguel Luengo-Oroz | Satellite Image Analysis in Humanitarian Contexts | | | satellite analytics | | | | |
| 52 | 68 | Dat Tien Nguyen, Shafiq Joty, Muhammad Imran, Hassan Sajjad, Prasenjit Mitra | Applications of Online Deep Learning for Crisis Response Using Social Media Information | 2016 | Nepal | Deep learning | Response | Crisis social media analytics | Retrospective validation using historical/archival data | Retrospective / historical data validation |
| 53 | 69 | Qikang Zhao; Xin Tan; Qingyan Meng; Liang Gao; Linlin Zhang; Xuewen Lei; Wenxu Shi; Mingming Zhu | Satellite and AI monitoring humanitarian crises in the Gaza Stripduring the early stage of Israeli–Palestinian conflict | 2024 | Gaza Strip | Deep learning (satellite analysis) | Response | Conflict damage assessment | Retrospective validation using historical/archival data | Retrospective / historical data validation |
| 54 | 70 | Nahian Ahmed, Adnan Firoze, and Rashedur M. Rahman. | Machine learning for predicting landslide risk of Rohingya refugee camp infrastructure. | 2020 | Bangladesh | Machine learning | Mitigation | Refugee camp safety planning | Retrospective validation using historical/archival data | Retrospective / historical data validation |
| 55 | 71 | Harish Kumar Chencharla Raghavendra. | AI-Enhanced Disaster Response Networks: A Framework for Resilient Communications. | 2025 | Global | Reinforcement learning | Response | Emergency communication networks | Conceptual / proposed framework | Conceptual / analytical guidance; no empirical validation |
| 56 | 72 | Clemens Havas; Lorenz Wendlinger; Julian Stier; Sahib Julka; Veronika Krieger; Cornelia Ferner; Andreas Petutschnig; | Spatio-Temporal Machine Learning Analysis of Social Media Data and Refugee Movement Statistics. | 2021 | Europe migration route | Deep learning | Preparedness / Response | Migration flow analysis | Retrospective validation using historical/archival data | Retrospective / historical data validation |

| | | | | | | | | | | |
|---|---|---|---|---|---|---|---|---|---|---|
| | | Michael Granitzer; Stefan Wegenkittl; Bernd Resch. | | | | | | | | |
| 57 | 73 | Wanxin Wu and Chun Pan. | Anomaly detection and early risk identification in digital disaster response-based on deep learning in public health. | 2025 | Global datasets | Deep learning (transformer -based) | Preparedness / Response | Public health emergency monitoring | Prototype / experimental model | Technical performance / model testing |

**Table S7:** Overview of grey literature (n=17)

| Sl. No | Ref. no. | Author / Organization | Year | Organization type | Geographic focus | Humanitarian context | AI / digital application | Deployment maturity | Evaluation / validation evidence | Governance, participation, or equity-related themes |
|---|---|---|---|---|---|---|---|---|---|---|
| 1 | 74 | Parkinson et al. / Humanitarian Leadership Academy & Data Friendly Space | 2025 | Humanitarian sector network | Global | Multi-sector humanitarian operations, displacement, climate crises | Generative AI, NLP, predictive analytics, geospatial analytics | Operational deployment / documented use case | Empirical descriptive / operational evidence | Privacy/data protection; accountability; bias/fairness; transparency; responsible AI; human oversight |
| 2 | 75 | CDAC Network | 2024 | Humanitarian communication network | Global | Conflict and crisis information ecosystems | Generative AI | Governance / analytical report without specific deployment | Conceptual / analytical guidance; no empirical validation | Information integrity/disinformation; accountability; protection of vulnerable groups; responsible communication |

| | | | | | | | | | | |
|---|---|---|---|---|---|---|---|---|---|---|
| 3 | 76 | Government of the United Arab Emirates | 2023 | National government | Global / UAE-linked | Disaster response coordination | Machine learning, geospatial analytics | Conceptual / proposed framework | Programme / strategy reporting without AI-specific validation | Digital coordination; policy framework; responsible digital systems |
| 4 | 77 | IFRC | 2024 | International humanitarian organization | Global | Migration and displacement | Digital platforms | Programme / strategy / guidance without specific AI deployment | Empirical descriptive / operational evidence | Digital inclusion/access; affected-community participation; protection of migrants and displaced populations |
| 5 | 78 | IFRC | 2012 | International humanitarian organization | Global | Disaster risk reduction and early warning | Digital early warning systems | Programme / strategy / guidance without specific AI deployment | Programme / strategy reporting without AI-specific validation | Community participation; early warning; disaster risk reduction |
| 6 | 79 | IFRC | 2019 | International humanitarian organization | Global | Climate-related disasters and disaster risk reduction | Machine learning, geospatial analytics | Programme / strategy / guidance without specific AI deployment | Programme / strategy reporting without AI-specific validation | Community-centred adaptation; local data; climate resilience |
| 7 | 80 | World Bank / TDRP | 2013 | International development organization | Global, including MENA examples | Post-conflict peacebuilding and DDR | Social media analytics | Programme / strategy / guidance without specific AI deployment | Conceptual / analytical guidance; no empirical validation | Conflict-affected populations; peacebuilding; digital information systems |

| | | | | | | | | | | |
|---|---|---|---|---|---|---|---|---|---|---|
| 8 | 81 | UNICEF | 2024 | UN agency | Global | Immunization equity and public health | Machine learning, geospatial analytics | Operational deployment / documented use case | Technical performanc e / model testing | Children; digital inclusion/access; public health equity; responsible data use |
| 9 | 82 | UNICEF ICT Division | 2024 | UN agency | Global | Humanitarian response, health, education, and child protection | Machine learning, data analytics | Operational deployment / documented use case | Empirical descriptive / operational evidence | Protection of vulnerable groups; children; responsible data use; privacy/data protection |
| 10 | 83 | IFRC / Hargrave | 2024 | International humanitarian organization | Global | Migration and displacement | Digital platforms | Programme / strategy / guidance without specific AI deployment | Empirical descriptive / operational evidence | Digital inclusion/access; affected-community participation; refugees, migrants and displaced populations |
| 11 | 84 | OCHA Centre for Humanitarian Data | 2024 | UN agency | Global | Disaster forecasting and anticipatory action | Machine learning, generative AI | Governance / analytical report without specific deployment | Conceptual / analytical guidance; no empirical validation | Responsible AI governance; accountability; bias/fairness; data protection |
| 12 | 85 | Humanitarian partners / Ukraine | Not specifie d | Humanitarian consortium | Ukraine | Cash assistance during conflict | Digital chatbot | Operational deployment / documented use case | Empirical descriptive / operational evidence | Conflict-affected populations; information integrity; digital access; protection |
| 13 | 86 | Qatar Computing Research Institute | 2019 | Research institute | Global / Qatar-linked | Disaster response and crisis monitoring | Machine learning, social | Operational deployment / documented use case | Empirical descriptive / operational evidence | Human-in-the-loop involvement; crisis informatics; operational decision support |

| | | | | | | | | | | |
|---|---|---|---|---|---|---|---|---|---|---|
| | | | | | | | media analytics | | | |
| 14 | 87 | UNHCR | 2025 | UN agency | Africa / Nairobi workshop | Climate-induced displacement risk | Machine learning | Pilot / limited field implementation | Empirical descriptive / operational evidence | Refugees/displaced populations; local data; climate displacement risk |
| 15 | 88 | UNHCR | 2024 | UN agency | Pakistan–Afghanistan | Refugee return monitoring | Natural language processing | Operational deployment / documented use case | Empirical descriptive / operational evidence | Refugees/migrants/displaced populations; legal status; local data; affected-community relevance |
| 16 | 89 | UNHCR | 2024 | UN agency | Global | Refugee protection and humanitarian assistance | Digital platforms | Programme / strategy / guidance without specific AI deployment | Programme / strategy reporting without AI-specific validation | Refugee protection; digital divide/access; legal status; data protection |
| 17 | 90 | WHO | 2020 | UN agency | Global | COVID-19 pandemic response | AI chatbots, digital health systems | Operational deployment / documented use case | Empirical descriptive / operational evidence | Public health emergency response; digital health access; risk communication |
